# Compounds from human odor induce attraction and landing in female yellow fever mosquitoes (*Aedes aegypti*)

**DOI:** 10.1038/s41598-022-19254-w

**Published:** 2022-09-21

**Authors:** Jan E. Bello, Ring T. Cardé

**Affiliations:** 1 Department of Entomology, University of California, Riverside, CA 92521 USA; 2 Present Address: Provivi Inc, 1401 Colorado Ave, Santa Monica, CA 90404 USA

**Keywords:** Entomology, Chemical ecology

## Abstract

The female *Aedes aegypti* mosquito is a vector of many human diseases such as yellow fever, dengue, and Zika. Transmission of these viruses occurs when an infected female mosquito locates a suitable human host, alights, and blood feeds. *Aedes aegypti* use human-emitted odors, as well as heat and visual cues, for host location. However, none of the previously identified human-produced compounds induce significant orientation and landing on a human host. Here we show that female yellow fever mosquitoes orient to and land on a mixture of compounds identified from human skin rubbings. Using odor collection, extraction, a two-choice, bioassay-guided fractionation, and chemical analysis, we identified mixtures of 2-ketoglutaric acid and L-lactic acid as landing attractants for female *Ae. aegypti.* The mixture of pyruvic acid and L-lactic acid were also found to be weakly attractive. Using ratio-response assays, we found that the attraction and alighting behaviors of the mosquitoes were directly related to the ratio of these compounds presented on the surface of the glass assay beads, suggesting that these compounds could mediate landing on a human host even at sub-nanogram dosages. The newly identified compounds fill a gap in our knowledge of odor-mediated attraction of *Ae. aegypti* and may lead to the development of new attractant-based mosquito control tactics.

## Introduction

*Aedes aegypti* mosquitoes are vectors of dengue, yellow fever, Zika viruses, and other dangerous pathogens. These pathogens are transmitted when infected female mosquitoes land and blood feed on human hosts. Mosquito attraction to a host can occur over many meters and at this range is thought to be mediated by a CO_2_ plume emanating from a host’s breath^1^. Carbon dioxide alone, however, rarely induces landing. Close to a host, perhaps within a meter or so, mosquitoes rely on host other odors, although heat and visual cues are also important for host seeking^1–4^. Landing on a host is a distinctive behavior which may be mediated by the same odors that mediate close-range attraction or by other odors that signify host identity. The blend of odors in human sweat, skin head space, and breath that are exploited by anthropogenic mosquitoes for host seeking has been analyzed using various chemical techniques^5–7^. Several studies have identified CO_2_, L-lactic acid, ammonia, short-chain carboxylic acids, short-chain saturated aldehydes, and 1-octen-3-ol as human-derived attractants for female mosquitoes^8–16^. However, typically these odors attract only a moderate proportion of mosquitoes over many minutes, suggesting that there missing components. Furthermore, it is unclear which human odors evoke close approach and alighting on the skin of a host and why certain individuals are more attractive than others^6,17,18^.

In wind-tunnel studies, female *Ae. aegypti* mosquitoes land on glass beads coated with human foot odor, which demonstrates attraction over at least a meter to human odors after exposure to CO_2_^19^. However, it is not known which compounds, or blends of compounds, from human foot odor induce these behaviors. Using collection and extraction of foot odors from an attractive subject using glass beads worn inside socks and shoes, fractionation guided by a two-choice bioassay, and chemical analysis, we isolated and identified components of human foot-odor extract that induce landing by host seeking *Ae. aegypti* mosquitoes. We also used ratio-response assays of these kairomones to show that doses of these compounds correlated with the attraction and landing rates of female mosquitoes, indicating that these compounds play a pivotal role in host selection behaviors.

## Results

### *Aedes aegypti* landing responses to human foot odor

**Figure 1:**
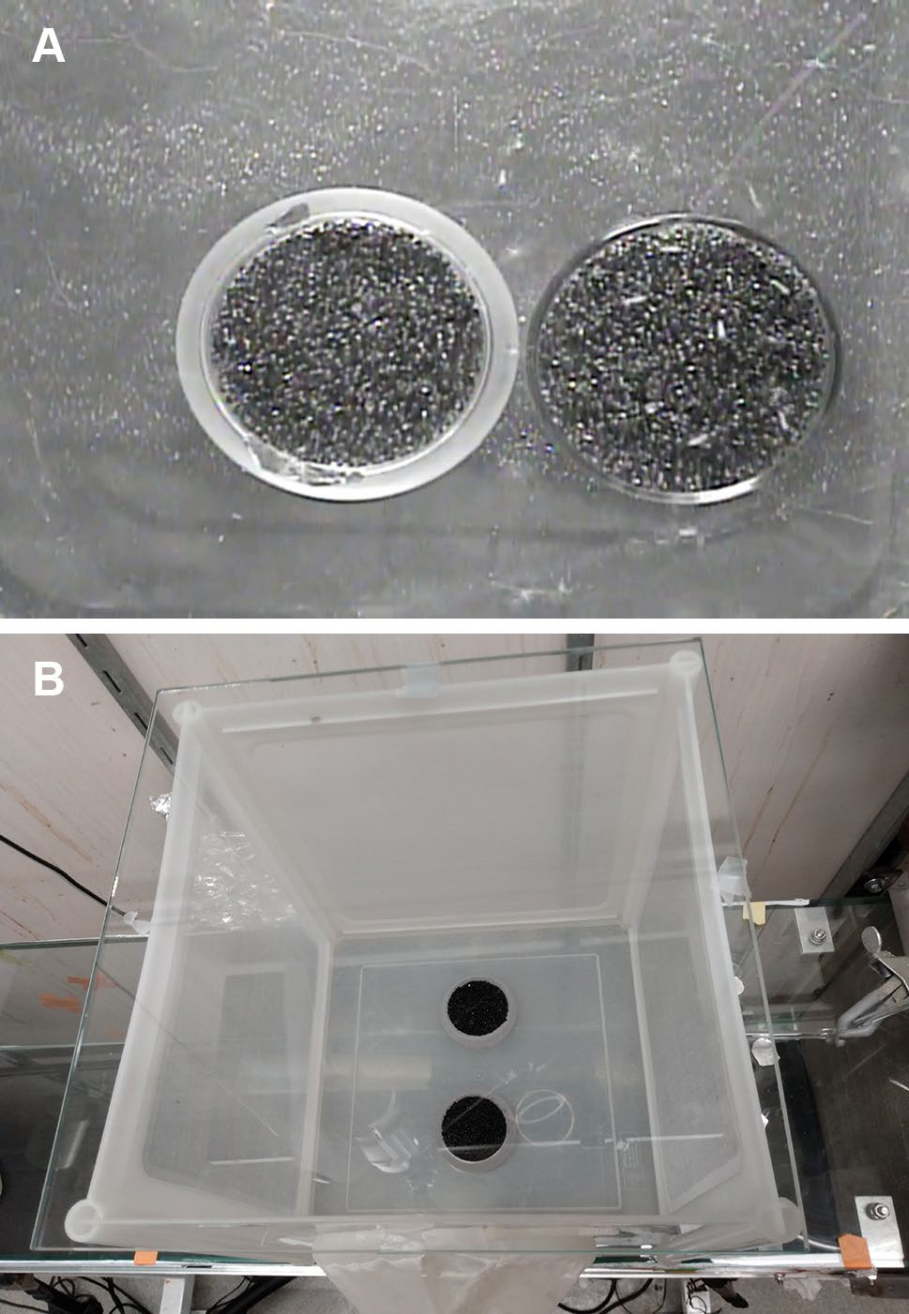
(**A**) Image of two-choice landing assay. Glass beads treated with an acetone extract of human foot odor are on the right with several female *Aedes aegypti* landed on the beads; solvent treated control beads are on the left. (**B**) Top view of the cage assay set-up including a 30 × 30 × 30 cm screen cage retrofitted with a glass top, two Petri dishes filled with black glass beads, and a glass tube attached to the CO_2_/air gas source.

**Figure 2:**
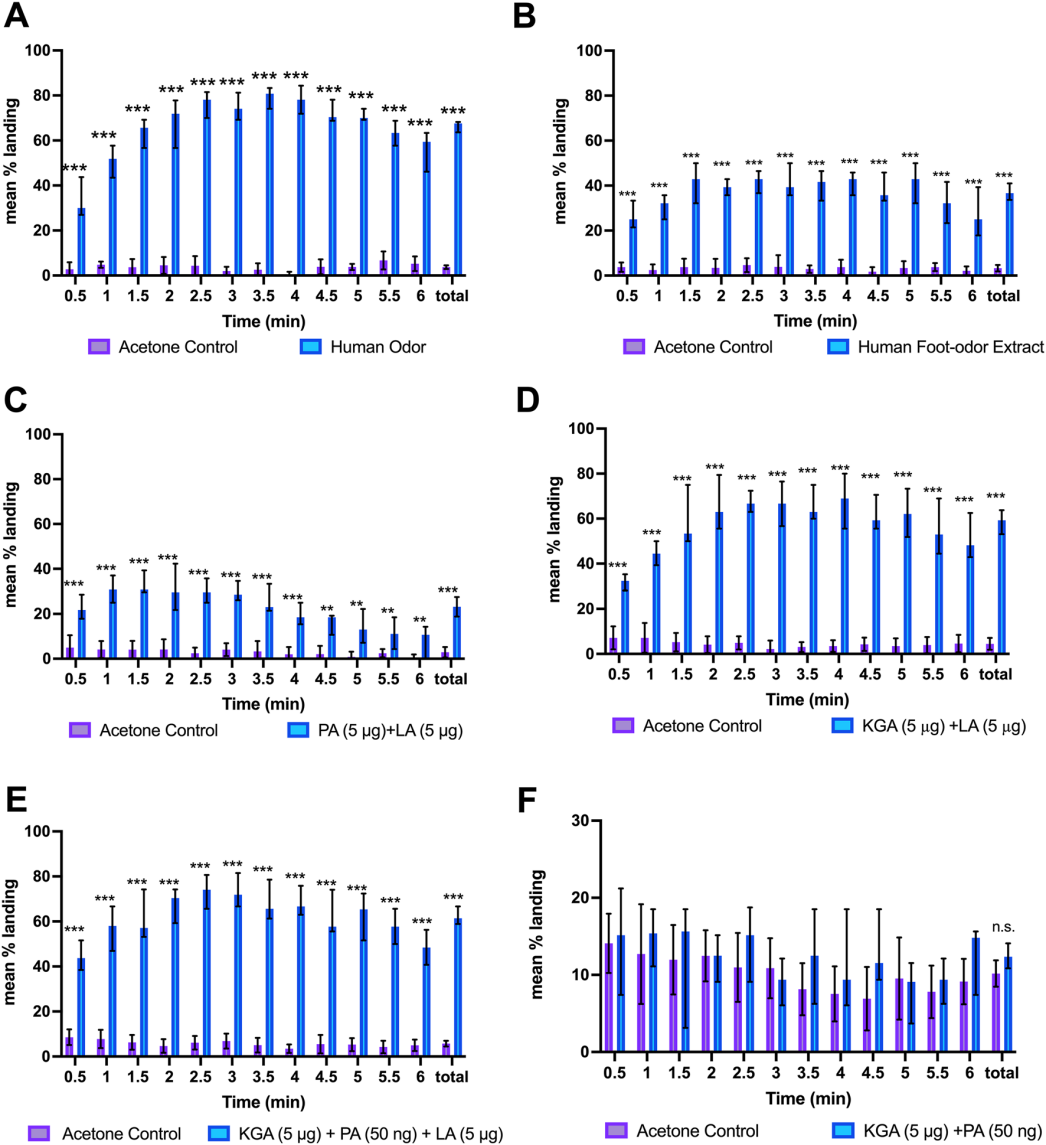
Landing response of host seeking *Ae. aegypti* to odor treated beads (n = 9 per treatment) (**A**) Mean % landing responses of female *Ae. aegypti* to beads used in odor collection containing unextracted human foot odor vs. solvent control over a 6-min period. Total landing responses to human foot odor treated beads were significant compared to control (*p* < 0.0001). Here and in subsequent panels, asterisks indicate significance in landing choice differences between two treatments (*** = *p* < 0.001, ** = *p* < 0.01, * = *p* < 0.05), error bars signify 95% CI. (**B**) Mean % landing responses of *Ae. aegypti* to human odor extract treated beads vs. solvent control over a 6-min interval. Total landing responses to human odor treated beads were significant compared to control. (*p* < 0.0001,). (**C**) Mean % landing of female *Ae. aegypti* to synthetic 2-component blend of pyruvic acid (PA, 5 µg) and lactic acid (LA, 5 µg). Total landing responses to synthetic 2-component blend were significant, but less than those seen from the 2-component blend of 2-KGA + LA (*p* < 0.0001). (**D**) Mean % landing responses of female *Ae. aegypti* to synthetic 2-component blend of 2-ketoglutaric acid (2-KGA, 5 µg) and lactic acid (LA, 5 µg). Total landing responses to synthetic 2-component blend were significant (*p* < 0.0001). (**E**) Mean % landing responses of female *Ae. aegypti* to synthetic 3-component blend of 2-ketoglutaric acid (2-KGA, 5 µg), pyruvic acid (PA, 50 ng), and lactic acid (LA, 5 µg) vs. solvent control. Total landing responses to synthetic 3-component blend were significant compared to solvent control (*p* < 0.0001). However, landing responses (**F**) Mean % landing of female *Ae. aegypti* to synthetic 2-component blend of 2-ketoglutaric acid (2-KGA, 5 µg) and pyruvic acid (PA, 50 ng). Total landing responses were not significantly different compared to control, indicating that lactic acid was necessary for activity. Note that the response levels to this blend are lower than to the other figures in this panel. To enable discernment of statistical differences the response scale was changed to a maximum of 30%.

Several studies have shown that female anthropophilic mosquitoes are attracted in wind-tunnel assays to human foot odors collected on glass surfaces^5,19–22^. With this in mind, we utilized a robust, two-choice bioassay modified from Webster et al.^22^ to determine if glass beads treated with human odor would elicit landing of female yellow fever mosquitoes in a small (30 × 30 × 30 cm) cage (Fig. 1A,B). In the assay mosquitoes would also be immediately and continuously exposed to an elevated level of CO_2_ for the entire bioassay interval, thereby ensuring bhost-seeking behavior. Although mosquitoes are attracted to all human odor, foot odor was utilized in this study due to its ease of collection, and the ability to collect large amounts of odor over an extended period. The worn foot odor scented beads elicited 66.8 ± 0.84 (SEM) % mean landing compared to 3.72 ± 0.27% mean landing on the control over the 6 min period (*p* < 0.0001, Fig. 2A, Video S1). Although we tallied landing over 6 min, it is noteworthy that there was substantial landing within the first 30 s sample interval (33.65 ± 2.21% mean landing on treatment vs 3.72 ± 0.27% mean landing on control). These results strongly implicated foot odor in short-range attraction and landing behaviors.

We used bioassay-guided fractionation to track the active compound to the polar fractions of the foot odor extract. Foot odors from the experimenter (JB) were collected on glass beads as described above, and the beads were extracted with various solvents. To simplify the chemical analysis of the extracts, only extracts from collected from JB were used. Subsequent bioassays indicated that acetone was more effective than hexanes and diethyl ether in extracting the odor (data not shown). The crude human foot-odor extract (HFE) induced mean landing rates of 36.9 ± 1.31% over a 6-min interval, compared to 3.27 ± 0.49% landings on acetone controls (*p* < 0.0001, Fig. 2B, Video S2). We then tried acid–base fractionation of the HFE extract. However, neither the acidic fraction nor the neutral residue induced landing rates significantly different than the controls (data not shown), suggesting that the landing cue(s) were either very water soluble and/or sensitive to acid or base treatment.

The HFE extract was then fractionated by normal phase-liquid chromatography on silica gel, eluting sequentially with pentanes, ethyl acetate, and methanol. The resulting three fractions were then assayed individually for their ability to induce landing in *Ae. aegypti* females. The pentanes and ethyl acetate fractions did not induce significant amounts of landing compared to the solvent controls, whereas the methanol fraction replicated the landing behaviors seen with the crude extract. (Video S3-S5) The MeOH fraction was further fractionated using column chromatography on silica gel, eluting with a stepwise solvent gradient of 100% EtOAc→100% MeOH, taking 85 fractions in total (Fig. S1). Fractions 66–67 induced 22.82 ± 2.29% mean total landing responses compared to 6.01 ± 2.52% mean landing on control (*p* < 0.01, Fig. S2A, Video S6), and fractions 73–80 induced landing at 34.15 ± 1.46% mean landing the 6- minute bioassay period, compared to 4.572 ± 0.67% mean landing on to the control treatment (*p* < 0.001, Fig. S2B, video S7). The results from these assays indicated that components of the short-range landing cue may be present in these active fractions.

### Identification of compounds stimulating landing from the active HFE fractions

**Figure 3:**
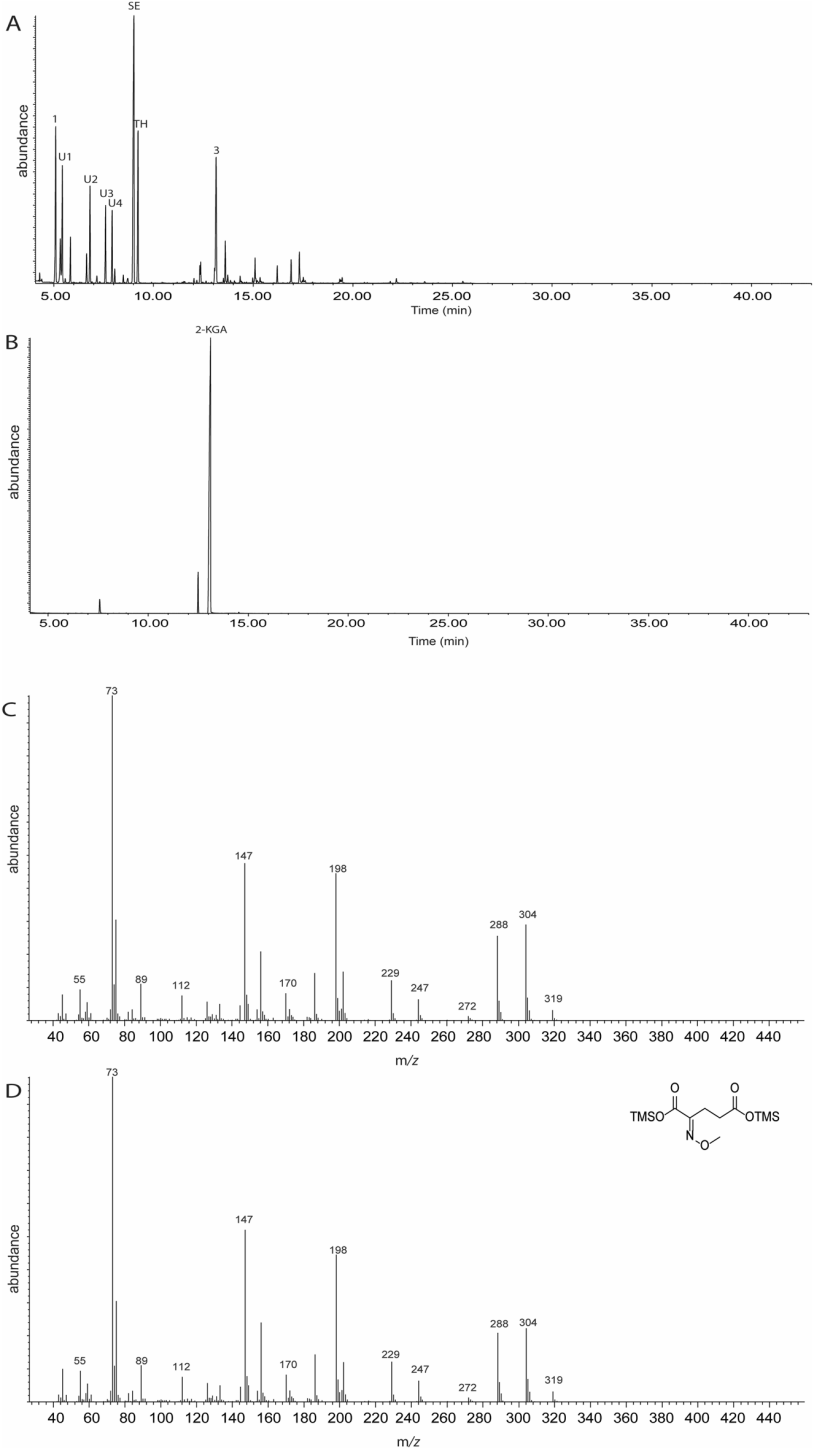
(**A**) GC chromatogram of methoxyaminated and BSTFA derivatized fractions 72–80 showing 8 major components. (**B**) GC chromatogram of derivatized 2-ketoglutaric acid standard. (**C**) Mass spectrum of compound 3. (**D**) Mass spectrum of derivatized 2-ketoglutaric acid standard.

On the assumption that the active compounds had to be at least semi-volatile in order to act over a distance, further analyses of the active fractions were done with coupled gas chromatography-mass spectrometry (GC–MS). In preliminary analyses of the two active fractions (combined 66–67, combined 73–80) no compounds were detected, suggesting that the compounds in the fractions were thermally degrading under the analysis conditions, or were extremely polar and so intractable to normal GC–MS conditions. Thus, the active fractions were derivatized to mask polar functional groups, first by methoximation to derivatize carbonyls, and then by silylation to mask alcohols, amines, and carboxylic acids. Analysis of the derivatized fractions by GC–MS (Fig. S2) showed that combined fractions 66–67 contained 4 major components and several trace components. The major components included derivatized lactic acid **1** (rt = 4.94 min, KI = 1132) and pyruvic acid **2** (rt = 5.88 min, KI = 1290), and two unknown components (compounds U1 and U2). The structures of lactic acid and pyruvic acid were confirmed by comparison of retention indices and mass spectra to those of synthetic standards subjected to the same derivatization protocols (Fig. S2). The two unknown compounds were also found in the inactive fractions 68–72, and active fractions 73–80 (Fig. 3), suggesting that they were less likely to be active, and that lactic and pyruvic acids were the main active compounds in fractions 66–67.

The derivatized fractions 73–80 showed 8 major components and several trace components (Fig. 3A). Four of the major components were identified as lactic acid **1** (rt = 4.95 min, KI = 1132), 2-ketoglutaric acid **8** (rt = 13.029, KI = 1757), serine (SR, rt = 8.67 min,*I* = 1404), and threonine (TH, rt = 8.89 min, KI = 1420). The remaining four major components (U1-U4) could not be identified. The structures of lactic acid and 2-ketoglutatic acid were confirmed by comparison of retention indices and mass spectra to those of derivatized synthetic standards (Fig. 3B–D). Thus, we focused further bioassay work on the identified components containing α-hydroxyacid and α-ketoacid moieties, based on the facts that these functional groups have been found in previously identified mosquito attractants^23,24^, and that these small molecules, although highly polar, are indeed semi-volatile, in contrast to the identified compounds serine and threonine, which are completely nonvolatile.

### Mixtures of the components identified from the active fractions induce short-range attraction and landing

To determine if mixtures of the compounds identified from the active fractions could induce landing and short-range attraction in host-seeking *Ae*. *aegypti*, we tested 2-component blends comprised of pyruvic acid (PA, 5 µg) + lactic acid (LA, 5 µg) and 2-ketoglutaric acid (2-KGA, 5 µg) + lactic acid (5 µg) in our cage landing bioassay. The mixture of PA + LA (5 µg each) induced 22.74 ± 1.56% mean overall landing over 6 min, compared to 2.92 ± 0.78% mean overall landing response with the acetone control (*p* < 0.0001, Fig. 2E, video S11). This result was comparable to the landing responses of host-seeking *Ae. aegypti* to the first bioactive fractions 66–67. The mixture of 2-KGA + LA (5 µg each) induced 58.19 ± 2.35% mean overall landing vs 4.47 ± 0.87% mean landings on the acetone control (*p* < 0.0001, Fig. 2D, Video S10).

To determine if individual components were able to induce landing, we bioassayed 2-KGA, PA, and LA individually at the same doses as in the previous assay. Lactic acid induced an overall landing response of 8.76 ± 0.792% compared to 4.92 ± 0.75% with the acetone control (*p* < 0.05, Fig. S2C, video S9), and 2-ketoglutaric acid induced an overall landing response of 6.76 ± 0.29% compared to 4.06 ± 0.34% for the acetone control (*p* < 0.01, Fig. S2D). Pyruvic acid alone did not induce significant landing when compared to acetone control (5.55 ± 0.22% vs 4.23 ± 0.38% respectively, see Fig. 2E). Thus, none of the individual components induced the much higher landing responses seen with the mixtures of LA + 2KGA or LA + PA, indicating strong synergism between the components of each of the 2-component mixtures.

### Ratio-response studies of landing cue components

**Figure 4:**
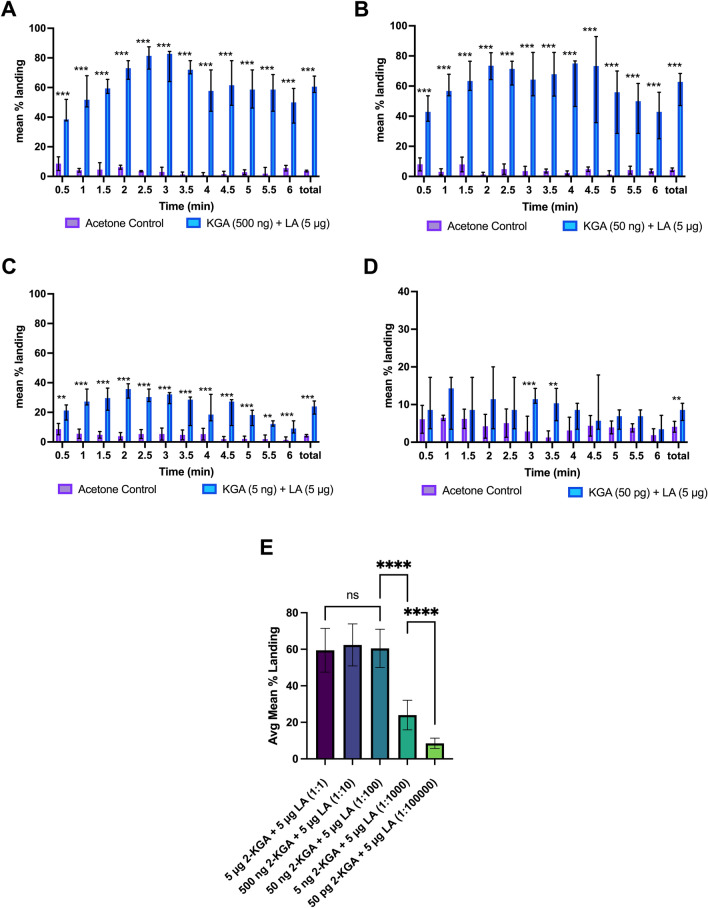
Ratio response assays to synthetic 2-component blend of 2-ketoglutaric acid and lactic acid (n = 5 per treatment). (**A**) Mean % landing responses of female *Ae. aegypti* on synthetic 2-component blend of 2-KGA (500 ng) and LA (5 µg). Total landing response to 500 ng/5 µg dose of 2-KGA + LA was significant compared to solvent control (*p* < 0.0001). Here and in subsequent panels error bars signify 95% CI. (**B**) Mean % landing responses of female *Ae. aegypti* on synthetic 2-component blend of 2-KGA (50 ng) + LA (5 µg). Total landing response to 1:100 ratio dose of 2-KGA + LA was significant compared to solvent control alone (*p* < 0.0001). (**C**) Mean % landing responses of female *Ae. aegypti* on synthetic 2-component blend of 2-KGA (5 ng) and LA (5 µg) (*p* < 0.0001). (**D**) Mean % landing responses of female *Ae. aegypti* on synthetic 2-component blend of 2-KGA (50 pg) + LA (5 µg) (*p* < 0.01). Total landing response to 50 pg/ 5 µg dose of 2-KGA + LA was significant compared to solvent control alone, but mean % landing decreased to levels similar to those seen in treatments of lactic acid alone (*p* < 0.01). Note that the response levels to this blend are lower than to the other figures in this panel. To enable discernment of statistical differences the response scale was changed to a maximum of 30%. (**E**) comparison of treatments using mixed ANOVA with Tukey’s multiple comparison tests indicating no significant difference between the landing response to 1;1, 1:10, 1:100 ratio of 2-KGA + LA. Decrease of the ratio of 2-KGA + LA to 1:1000 resulted in a significant decrease in total mean landing response, indicating that the ratio of these compounds is important to biological activity (*p* < 0.0001). Decrease of the ratio of 2-KGA + LA to 1:100,000 led to another significant decrease in landing response as compared to the 1:1000 ratio dose, suggesting that as the ratio of these components decrease, the behavioral response also decreases (*p* < 0.0001).

To determine the effect of the ratio between the landing cue components, we bioassayed two-component blends of 2-KGA + LA and PA + LA over a range of ratios. In a first test, four doses of 2-KGA were tested (e.g., 500 ng, 50 ng, 5 ng, and 50 pg) in a blend with 5 µg LA, resulting in a 1:10, 1:100, 1:1,000, and 1:100,000 ratio of 2-KGA + LA in the varying treatments. The amounts of lactic acid were left constant at 5 µg because lactic acid is the largest component of the human sweat exacts and was found in both active and inactive fractions of the HFE, suggesting that variation to the amounts of LA in the blends may not be as important to attraction as variation in the amounts of PA or 2-KGA. The 500 ng 2-KGA + 5 µg LA (1:10 ratio) and 50 ng 2-KGA + 5 µg LA (1:100) dosages elicited averages of 62.39 ± 2.08% and 60.52 ± 3.69% mean landing responses over the 6-min interval, compared to solvent controls, which elicited mean landing responses at 3.65 ± 0.25% and 4.47 ± 0.52% respectively (*p* < 0.00001 and *p* < 0.00001 respectively, Fig. 4A,B). Treatment with the blend containing 5 ng 2-KGA (1:1,000) produced mean landing responses to 23.99 ± 1.52% compared to 4.25 ± 0.35% in the solvent control (*p* < 0.00001, Fig. 4C). To identify the threshold for activity the dose of 2-KGA was decreased 100-fold further to 50 pg (1:100,000), which resulted in 8.53 ± 0.84% mean overall landings compared to 4.10 ± 0.65% on solvent control (*p* < 0.05, Fig. 4D). The results of the dose/ratio response assays, along with the results original 5 µg 2-KGA + 5 µg LA assay, were compared using a mixed model ANOVA with Tukey’s multiple comparison test to determine significance between the different dosages. The landing responses of female *Ae. aegypti* to the 5 µg (1:1), 500 ng (1:10), and 50 ng (1:100) 2-KGA + 5 µg LA doses were not significantly different, indicating that these range of dosages were equally effective (Fig. 4E). Lowering the dosage to 5 ng 2-KGA + 5 µg LA (1:1,000) resulted in a significant drop in the landing response from the 1:100 dosage (*p* < 0.0001, Fig. 4E). Lowering the dosage even further to 50 pg 2-KGA + 5 µg LA (1:100,000) again resulted in a significant decrease in landing compared to the 1:1000 dosage of 2-KGA + LA (*p* < 0.0001, Fig. 4E).

**Figure 5:**
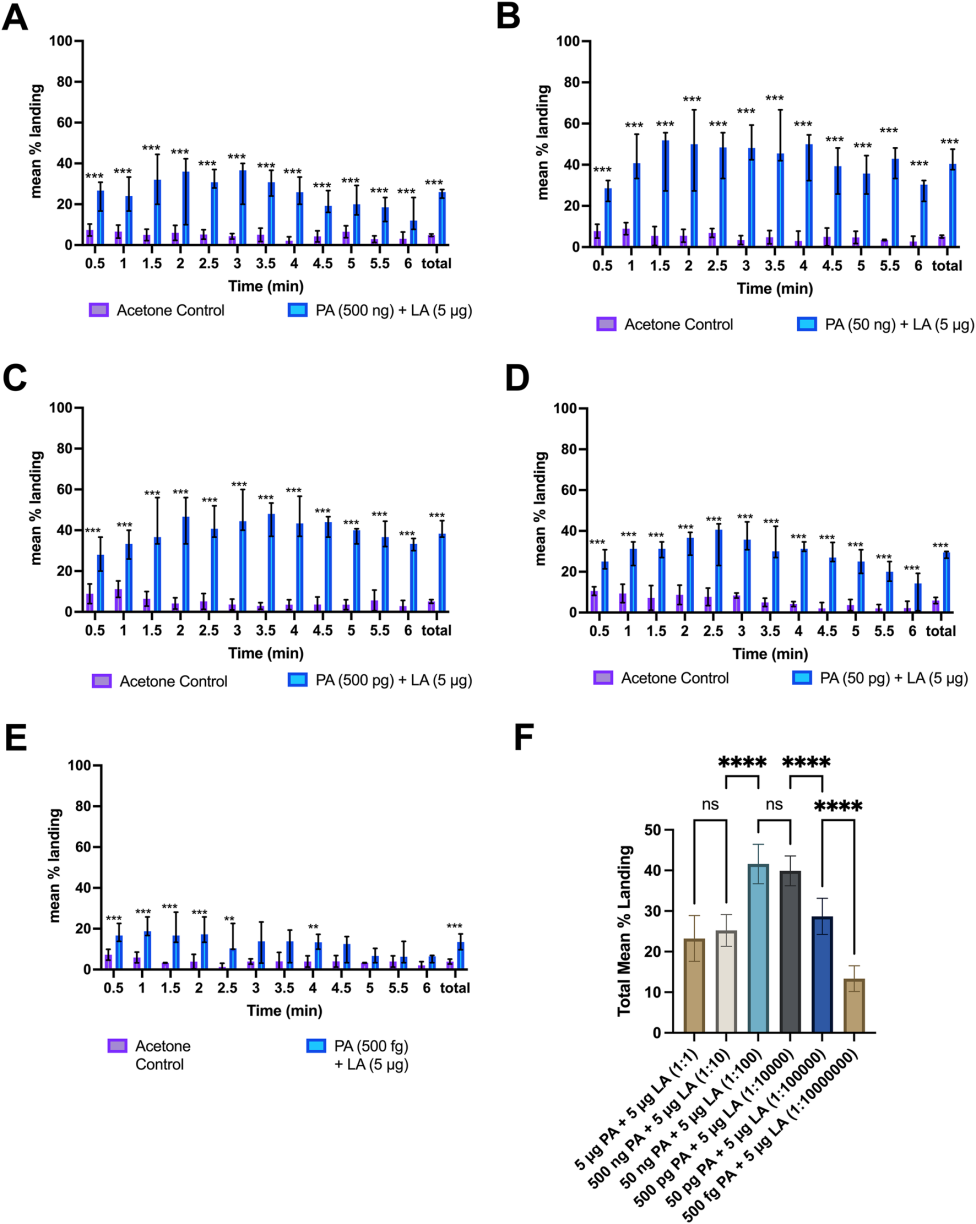
Ratio response assays to synthetic 2-component blend of pyruvic acid and lactic acid vs. solvent control (n = 5 per treatment). (**A**) Mean % landing responses on 2-component blend of 500 ng + 5 µg dose of PA + LA (1:10). Total landing response to 500 ng/ 5 µg dose of PA + LA was significant compared to control (*p* < 0.0001). In all panels error bars signify 95% CI. (**B**) Mean % landing responses on 2-component blend of PA (50 ng) + LA (5 µg). Total landing response to 50 ng + 5 µg dose of PA + LA (1:100) was significant compared to control alone (*p* < 0.0001). (**C**) Mean % landing responses 2-component blend of 1:10,000 PA + LA (500 pg PA + 5 µg LA). Total landing response to 1:10,000 ratio of PA + LA was significant compared to control alone (*p* < 0.0001). (**D**) Mean % landing responses on 2-component blend of 1:100,000 PA + LA (50 pg PA + 5 µg LA). Total landing response to 1:100,000 ratio of PA + LA was significant compared to control but mean % landing decreased to levels like those in the highest doses [5 µg (1:1) and 500 ng (1:10)] of PA + LA (*p* < 0.001). (**E**) Mean % landing responses on 2-component blend of PA (500 fg) and LA (5 µg). Total landing response to 500 fg/ 5 µg dose of PA + LA was significant compared to control alone but mean % landing decreased to levels like those seen in treatments of lactic acid alone (*p* < 0.001). (**F**) Comparison of treatment groups using mixed ANOVA with Tukey’s multiple comparison tests. Results of 1:1 and 1:10 ratio of PA + LA were not significantly different, while decreasing the ratio to 1:100 to 1:10,000 resulted in a significant increase in landing response (*p* < 0.0001), decreasing the ratio of PA + LA to 1:100,000 resulted in a decrease in response similar to those seen in the higher dosage ratios (1:1 and 1:10) of PA + LA. Decreasing the ratio of PA + LA to 1:10,000,000 resulted in a significant decrease in response seen in the 1:100,000 ratio dose (*p* < 0.0001).

We then bioassayed two-component blends of PA and LA at various ratios of PA:LA to determine if the landing behaviors seen on the 1:1 PA + LA blend were ratio dependent. To determine the optimal doses and ratio of PA to LA, five different dosages of pyruvic acid were tested e.g. (500 ng, 50 ng ,500 pg, 50 pg, and 500 fg) with a constant amount of LA set at 5 µg for the same reasons as described above, resulting in a 1:10, 1:100, 1:10,000, 1:100,000, and 1:10,000,000 PA to LA treatments respectively. These dosages were chosen to determine to optimal ratio of PA:LA for the highest landing activity, and to determine the lowest possible ratio of PA to LA needed for landing to occur. 1:10 PA + LA (500 ng PA + 5 µg LA) resulted in 25.24 ± 1.08% mean total landings over the course of the assay compared to 4.14 ± 0.622% on control (*p* < 0.0001, Fig. 5A). The treatments utilizing 1:100 PA to LA (50 ng PA + 5 µg LA) resulted in an increased landing response of 41.59 ± 3.81% on the treated petri dish compared to 5.11 ± 0.67% landings on control (*p* < 0.0001, Fig. 5B). Decreasing the treatment ratio to 1:10,000 (500 pg PA + 5 µg LA) led to 39.73 ± 1.55% total mean landings, while mean total landings on solvent control was 5.12 ± 0.42%, (*p* < 0.0001, Fig. 5C). When the ratio of PA to LA was lowered to 1:100,000 (50 pg, 5 µg LA) the mean total landing was 28.82 ± 0.60% compared to 5.9 ± 0.69% on the control (*p* < 0.0001, Fig. 5D). When the ratio of PA:LA was lowered even further to 1:1,000,000 (500 fg PA, 5 µg LA) the mean total landing response decreased to 13.34 ± 1.26% compared to 3.89 ± 0.54% mean total landing response on the solvent control (*p* < 0.05, Fig. 5E). The results of the PA + LA dosage studies were also compared using a mixed model ANOVA with Tukey’s multiple comparisons test to determine significance between the treatment groups. The landing response of the female *Ae. aegypti* at the 10:1, 1:1, and 1:10 dosages of PA + LA were not significantly different. Decreasing the dosage to 1:100 PA + LA resulted in a significant increase in response compared to the 1:10 dosage (*p* < 0.0001). However, there was no significance difference between the response seen in the 1:10,000 dosage of PA + LA compared to the 1:100 dose. There was a significant lowering of response when decreasing the dosage to 1:100,000 PA + LA when compared to the 1:10,000 and 1:100 doses (*p* < 0.0001), indicating an optimal ratio of PA + LA results in increased activity. At the lowest dosage of 1:10,000,000 PA + LA (500 fg PA + 5 µg LA) the landing response is again significantly decreased compared to the next highest dosage (*p* < 0.0001, Fig. 5F).

### Lactic acid is required for bioactivity of the blends, but a combination of all three (LA + PA + 2-KGA) components are not synergistic and does not result in increased bioactivity

To determine if the three known components in the active fractions were able to induce a synergistic effect on *Ae. aegypti* landing, a blend of 2-KGA (5 µg, 34 nmol) + PA (50 ng, 5.6 nmol) + LA (5 µg, 55 nmol) was bioassayed. This ratio was used based on the results from the 2-component dose–response experiments, where these ratios of LA to either PA or 2-KGA induced the highest mean percent of landings. The natural ratio of pyruvic acid to lactic acid in human sweat has been reported to be between 1:100 to 1:1000, and this ratio was also taken into consideration while making this blend^25^.

The synthetic blend of all three components induced 62.21 ± 1.28% mean percent landing vs. 5.73 ± 0.42% on the acetone control (*p* < 0.0001, Fig. 2C, Video S8). The results of the 3-component blend were compared to the response *of Ae.* aegypti females to the two comparable 2-component blends of 2-KGA + LA (1:1) and PA + LA (1:100) using a mixed model ANOVA followed by Tukey’s multiple comparison test to determine if there were any significant differences between the response to these treatments. When comparing the 1:100:1 ratio 3-component blend to the 1:1 ratio 2-KGA + LA blend, there was no significant difference between the response treatments indicating that the combination of the three components in their optimal ratios did not induce an increased landing compared to the 2-component blend of LA + 2-KGA (Figure S3F).

In a final test, we bioassayed a 2-component blend of PA + 2KGA (50 ng PA, 5 µg 2-KGA). The mixture of PA + 2-KGA induced 12.39 ± 0.53% mean landing compared to 10.18 ± 0.57% mean landing in the acetone control, which was not significantly different (Fig. 2F). The lack of significant response to the 2-component blend of PA + 2-KGA compared to the 3-component blend with the same quantities of PA and 2-KGA verified that LA is a crucial component of the blend needed to induce high levels of landing in host-seeking yellow fever mosquitoes.

## Discussion

The attraction of *Ae. aegypti* and other anthropophilic mosquitoes to human odors is well established, but the specific human odors that induce short-range orientation and alighting behaviors were previously unknown^1^. Previously identified human-emitted odors such as lactic acid, various carboxylic acids, ammonia, ketones, various alcohols, aldehydes, and amines induce some mosquito attraction, especially when presented with CO_2_ and assayed for several minutes^9–12^. In the work described here, we identified two highly polar components of the human odor blend that, in combination with lactic acid, induce landing in female *Ae. aegypti*. Using bioassay-guided fractionation, microscale derivatization, and a series of two-choice landing assays we determined that 2-component mixtures of lactic acid (LA) and 2-ketoglutaric acid (2-KGA), and lactic acid and pyruvic acid (PA) rapidly induce orientation and landing at physiologically relevant doses. The two-choice landing assay was able to quickly and clearly establish preference of host-seeking *Ae. aegypti* to odor treatments (both synthetic and natural extracts) compared to controls^26^. *Aedes aegypti* is an opportunistic or “quick to bite” species, and behavioral assays which measure rapid responses to test odors at physiologically relevant concentrations should be more indicative of natural responses than assays that might require many minutes of odor exposure for response, such as olfactometer bioassays^26^. Lactic acid and pyruvic acid are known human sweat components and individually, have been shown to be weakly attractive to mosquitoes^4–8,23,25–28^. However, to our knowledge, this is the first report of 2-ketoglutaric acid as a short-range attractant for yellow fever mosquitoes. 2-Ketoglutaric acid is a metabolite from the citric acid cycle and has been found in metabolic analyses of human sweat, urine, blood, saliva, and may also be a metabolite from normal skin flora^29–31^. Although our assays clearly indicated that mixtures of 2-KGA + LA or PA + LA induced short-range orientation and alighting in host-seeking *Ae. aegypti* after CO_2_ exposure, none of these components induced landing individually. The mixture of PA + 2-KGA also did not elicit significant levels of landing. These results suggest that lactic acid is a key factor in the mosquito’s preference for human odor, but it is not very active alone: in our assay LA must be presented with PA and 2-KGA to elicit high levels of landing.

Both the dosage and ratios of odorants are likely to be important for the attraction of host seeking *Ae. aegypti*. We showed that the landing responses of *Ae. aegypti* females to LA + 2-KGA treatments decreased with decreasing amounts of 2-KGA, whereas the landing responses to PA + LA treatments were optimal in treatments where the ratio of PA:LA resembled those seen naturally occurring in human sweat (1:100–1:1000)^25^. Landing responses decreased as the ratio of PA to LA decreased to 1:100,000 PA + LA (50 pg PA + 5 µg LA). The variation in landing responses in our assays testing different ratios of PA to LA indicated that changes in the ratios of these two human odor components may be a factor in the variable attraction of different human hosts to mosquitoes^17,18^. Evaluation of a mixture of all three identified components (2-KGA + PA + LA) at the optimal ratios found in the landing assays induced mean % landing responses equivalent to those seen with the 2-component 2-KGA + LA treatments, indicating that although pyruvic acid is present in the human odor extracts, it is not essential for short-range orientation and landing. Although less active than 2-KGA in our short-range assays, PA may be important for longer-range attraction of mosquitoes to a host, as 2-ketoglutaric acid is less volatile than PA and may be only active as a short-range cue for landing and attraction. Compared to 2-KGA, pyruvic acid is much more volatile and is also a source of acetone, ethanol, and acetic acid as it degrades in air. The decomposition of pyruvic acid may also be a source of endogenous CO_2_, activating CO_2_-sensing neurons in the maxillary palps of *Ae. aegypti* and initiating alighting when mosquitoes are no longer in the plume of CO_2_ emanating from human breath^27^. The mixture of 2-KGA + LA also induced probing after landing when tested at the optimal ratio (video S12), further indicating that 2-KGA + LA signals a suitable host for a blood meal.

It remains to be determined whether other human-emitted compounds attractive to *Ae. aegypti* might enhance the attractiveness of a blend of 2-KGA and lactic acid. The attractiveness of odor blends can involve simple additive effects, synergism and redundancy. Some compounds can increase or decrease attractiveness of a blend dependent on the quantity added^32^. Some human odors such as 6-methyl-5-hepten-2-one (sulcatone) can be antagonistic to attraction^33^. In *Ae. aegypti,* CO_2_ combined with lactic is a weak attractant. Carbon dioxide with the same blend and dose of KGA and lactic acid on glass beads found in the present trials to mediate landing and probing also evokes upwind flight and landing on the odor source over 1 m in a wind tunnel^34^. The possibly that KGA also mediates attraction in other *Aedes* species and mosquitoes remains to be studied.

Geier et al.^35^ found that CO_2_ plus a blend of ammonia, and individual C(1)-C(3) and C(5)-C(8) carboxylic acids induced upwind displacement of *Ae aegypti* in a Y-tube olfactometer. A commercial lure (BG Sentinel) emitting lactic acid, hexanoic acid and ammonia with carbon dioxide is widely deployed in surveillance traps^36^. Field trapping studies found that nonanal and decanal, also human released, with CO_2_ were attractive individually and more attractive as a blend. Curiously, the two aldehydes suppress trap catch of an otherwise attractive hexanoic acid bait^32^. Generally, the attractiveness of a blend can vary widely due to dispenser dose and component ratio.

Future work seeking to establish whether a blend of KGA and lactic acid is rendered more attractive by the addition of the other reported attractants can rely on empirical screening in wind tunnel, landing and field-trapping assays. To determine whether individual compounds are used in combination in natural orientation to a human host will need to ensure that odors are assayed in physiologically and ecologically relevant concentrations and ratios and utilize physiologically relevant levels of CO_2_, This approach might finally answer the decades-old question as to why humans vary in their attractiveness to *Ae. aegypti*^6,33^.

The identification of 2-KGA and PA as landing cues for *Ae. aegypti* mosquitoes from human foot odor should open numerous new avenues for research. For example, identification of the sensilla responsible for detection of these odors and the relevant odorant binding receptors will reveal how these compounds are processed in the olfactory system. Structure–activity relationship studies can then probe the structural motifs that underpin the bioactivity of these compounds. Studies on whether 2-KGA and PA can be detected by mosquitoes as long-range attractants would establish if these compounds can be utilized for surveillance and/or mosquito management.

Furthermore, *Ae. aegypti* has diverged into two ecologically distinct populations, the zoophilic *Ae. aegypti formosus* and the anthropophilic *Ae. aegypti aegypti*^37^. The preference of *Ae. a. aegypti* for human hosts is a recently evolved trait that may have occurred more than once from the forest dwelling, ancestral *Ae. aegypti formosus*^37^*.* The cause of the variation in the host odor preferences between these two distinct populations of yellow fever mosquitoes is san open question. Thus, it will be informative to determine whether the short-range attractants identified in our study can be detected by both populations of *Ae. aegypti*, or whether one or more of 2-KGA, PA, and LA are specific odor cues which signify a human host only for the anthropophilic *Ae. ae. aegypti*.

## Methods

### Insects

A colony of *Aedes aegypti*, Orlando strain, was maintained in under L:D 14:10 at 27 °C and 70% RH. The colony was propagated with bovine blood (Hemostat Laboratories, Dixon CA, USA) through a heated membrane feeding system. Carbon dioxide was provided by exhaled breath to facilitate feeding. Larvae were reared in plastic containers filled with deionized water and fed Tetramin® fish food (Tetra, Blacksburg VA, USA). Pupae were collected into plastic bowls and the bowls were held in screen cages (30 × 30 × 30 cm, BugDorm-1, Megaview Science Co. Ltd. Talchung, Taiwan) until eclosion and then provided a diet of 10% sucrose solution in deionized water after emergence. Emerged males and females were kept together in the screen cages, and the females used in the bioassays were assumed to have mated prior to the experiments. Females were used in experiments 5–14 d post-eclosion and were not blood fed. Approximately 12–14 h prior to experiments, cohorts of mosquitoes were starved and held in a light box in the assay room with the same light and humidity conditions as the rearing rooms (27 °C and 50–70% RH). Thirty min prior to testing, 27–35 female mosquitoes were transferred to a clean screen cage (30 × 30 × 30 cm) fitted with glass panels on top to allow for viewing, to acclimatize prior to the assays. The assay room was maintained at 27 °C and 70% RH, and the assay room was kept dark apart from a 940 nm infrared light source illuminating the assay cage, and a single lamp with a 40 W light bulb facing away from the assay cage.

### Odor sources

Human foot odor was collected using 2-mm black glass beads (Darice Inc. Strongsville OH, USA) using standard odor collecting techniques^5,19,21^. Beads were cleaned by soaking in a solution of deionized water and 10% detergent (Micro 90) for 1 h, then maintained in the soaking solution and sonicated for 1 h. The beads were then thoroughly rinsed with deionized water, dried, rinsed twice with acetone (ACS grade, Fisher Scientific, Waltham MA, USA), and heated to 250 °C for ~ 12 h. For initial scented bead assays foot odor was collected on black glass beads (80-100 g) worn in a cotton socks(Haynes Inc., Winston-Salem, NC, USA) inside the shoes of the experimenter (JB) and two “attractive” subjects for 4 h. The scented beads were used immediately after collection in the assays. For the subsequent assays beads (80–100 g) were worn in cotton socks and shoes by the experimenter (JB) for approximately 4 h prior to extraction. The socks were previously cleaned using unscented detergent and the feet of the experimenter were washed with unscented soap prior to collection. Only the odors collected from the experimenter were used in the subsequent assays to simplify the chemical analysis of compounds.

### Odor extraction

The odors from the glass beads were extracted by soaking the beads in acetone (2 × 120 mL, Optima grade, Fisher Scientific) and sonicating for 30 min at room temperature. Acetone was chosen due to its ability to extract both polar and non-polar compounds efficiently, its stability over long periods of time, and its high volatility as compared to other solvents, allowing for quick evaporation. The combined acetone extracts were filtered through a glass wool plug, transferred to a 500 mL round-bottomed flask, and concentrated by rotary evaporation to approximately 40 mL. The concentrated extract was transferred to two 20 mL glass scintillation vials and further concentrated to 10 mL under a flow of nitrogen gas. The odor collection and extraction procedures were repeated for 15 consecutive days to collect enough material for the bioassay-guided fractionation procedures, and derivatization reactions that were required for structural elucidation of active compounds. The concentrated extracts were combined (approx. 150 mL) and further concentrated to 50 mL (90 h total collection) to be used in the subsequent fractionation and identification steps.

### Odor treatment

For each replicate, clean 2-mm black beads as described above (8–10 g) were placed in a clean glass Petri dish (7 cm diam.) and 500 µL of foot odor extract (approx. ~ 90 min of collection) was transferred dropwise evenly onto the beads using a 1 mL disposable syringe with a 24-gauge needle. A second Petri dish was prepared and treated in the same manner with 500 µL of clean acetone as the control. The treatment and control Petri dishes were then placed under a fume hood for 20 min to allow for the solvent to evaporate fully prior to assays, before being placed in the bioassay cages (see below).

### Landing assays

We released 27–35 female mosquitoes into screen cages (30 × 30 × 30 cm) fitted with glass panels on top to allow for viewing and recording, 30 min before experiments, and the cages were moved to an assay table and left undisturbed. Two Petri dishes, containing the odor extract and control treatment respectively, were placed in the center of the cage 3 cm apart. To introduce CO_2_, a glass tube (85 × 6 mm i.d.), connected via tygon tubing to an air/CO_2_ flow controller and gas supply, was positioned 5 cm to the center of right side of the screen cage. (Without added CO_2_, mosquitoes would not orient to the stimuli.) The air/CO_2_ mixture was turned on, the experimenter left the room, and the landing behavior was recorded for 6 min with a video camera. The CO_2_ concentration was maintained at 4% by mixing medical grade air (Air Source Gas, Long Beach, CA) and pure CO_2_ using flow meters. The 96% air/ 4% CO_2_ mixture (1 L/min) was blown into the screen cage. Nine replicates of 27–35 mosquitoes were carried out for each treatment. For the ratio-response assays 5 replicates of 27–35 mosquitoes were carried out for each treatment ratio. To prevent contaminating the cages with skin odors, the experimenter wrapped his arms in Press and Seal® plastic wrap (Glad, Johnson and Johnson) and always wore nitrile gloves during the assay. To ensure responses of the mosquitoes were from the introduction of the air/CO_2_ gas mixture to the assay cage, and not CO_2_ from a human source, the experimenter held his breathe before entering the assay room to introduce the odor-treated Petri dishes and to turn on the air/CO_2_ mixture, until exiting the room once the bioassay was started. The mosquitoes were then immediately and continuously exposed to an elevated level of CO_2_ for the whole bioassay interval. Landing responses were measured by calculation of the mean landing percentage:

$$\left( {\left( {\# of\,mosquitoes\;landed \div \;\# total\;of\;mosquitoes\;released\;in\;the\;arena} \right) \times 100.} \right)$$

every 30 s for 6 min and then calculating an average of these 30-s mean landing percentages to obtain the total mean landing over the 6-min bioassay interval.

### Determination of attraction

Attraction was determined by the landing rates of female *Ae. aegypti* on worn beads presented in the previously described landing assays^34^. Individuals whose odors induced high overall mean landing percentages (> 50%) over the course of the assay were considered highly attractive, individuals whose odors induced mean overall landing between 30 to 49% were deemed attractive, and those who induced mean overall landing of < 29% were deemed somewhat attractive. Of the six individuals tested, three were deemed highly attractive including the experimenter (JB). However to simplify the chemical analysis of the odor extracts, only odors from the experimenter were collected and used for the subsequent bioassays.

### Video recording

Videos of the landing assays were recorded with a CCTV video camera (ICD 48, Ikegami Electronics USA, Inc., Maywood NJ, USA) with 6-mm lens. Cameras were placed 1.1 m directly above the glass ceiling of the assay cage to allow for clear viewing of the bioassay arena. Video was recorded in audio–video-interwave (AVI) format onto an external hard-drive at 30 frames/sec using Noldus video capture software (Noldus MPEG recorder, Noldus Information Technology BV, Wageningen, The Netherlands). Video was converted to MP4 format to compress the video size using Handbrake open-source video transcoder (downloaded from https://handbrake.fr/ 03/01/2020).

### Data analysis

Video files were analyzed (blind) by counting the numbers of mosquitoes on the black beads every 30 s for the 6 min duration of the experiment. Differences in numbers of mosquitoes per treatment were compared using a paired t-test with statistical significance determined using the Bonferroni-Dunn method, with alpha = 0.05. Comparisons between treatments were performed using mixed model ANOVA with Tukey’s multiple comparison test with experiment-wise alpha set to (0.05, 95% confidence interval). All analyses were carried out with Graphpad Prism 8.0.

### Fractionation of extracts

Concentrated odor extracts were fractionated into non-polar, semi-polar, and polar fractions using column chromatography. Five mL of the concentrated foot odor extract (equivalent to 8 h of collection) was concentrated to 0.5 mL under a stream of N_2_ gas and was loaded onto a 12.7-cm pipette column containing a 6 cm long bed of silica gel (10 g, 230–400 mesh) equilibrated with pentane. The column was then eluted with 5 mL of pentanes (Optima Grade, Fisher Scientific), followed by 5 mL of ethyl acetate (EtOAc, Optima Grade, Fisher Scientific), and finally 5 mLof methanol (MeOH, HPLC Grade, Fisher Scientific). The column was again washed with 5 mL MeOH to ensure that all the compounds had been eluted. Each fraction was concentrated to 1.5 mL under a flow of N_2_ gas, and each fraction was tested for bioactivity using the previously described biological assays (500 µL of each fraction). The remaining active methanolic fractions (~ 2 mL) along with the remaining extract (40 mL, ~ 64 h of collection time), were then combined, concentrated to 5 mL, and fractionated using flash column chromatography (40 mm ID column, 120 g silica) with a stepwise gradient solvent system eluting sequentially with 200 mL each of EtOAc, 75% EtOAc/25% MeOH, 50% EtOAc/50% MeOH, 25% EtOAc/75% MeOH, and 100% MeOH. Fractions of ~ 12 ml were taken, resulting in ~ 16 fractions per solvent concentration, and 85 total fractions. Each fraction was concentrated to 5 mL, and then 500 µL (equivalent to ~ 7 h of collection time) of each fraction was tested for bioactivity using the previously described bioassays. The bioassays utilized the corresponding solvent mixture as control. The active fractions were then combined with adjacent active fractions, concentrated, and then analyzed by GC–MS to identify candidate active compounds that induced landing by *Ae. aegypti* females.

### Identification of compounds in active fractions

Preliminary bioassays indicated that 12 fractions (Fraction 66–67 & 73–80) induced landing by *Ae. aegypti* females. One mL aliquots of each fraction group were concentrated to 10–20 µL under a stream of N_2_ and analyzed via GC–MS. All samples were analyzed on an Agilent 6890 gas chromatograph coupled to an Agilent 5975C mass spectrometer operated in electron impact (EI) mode. The GC was fitted with a HP-17MS column (30 m × 0.25 mm × 0.25 µm film thickness) and the temperature program used was 60 °C for 1 min, then increased 10 °C/min to 280 °C, and held at 280 °C for 20 min. Initial analyses of the active fractions showed no compounds, suggesting that the highly polar components in the extracts were not eluting from the column, or were being destroyed by the high temperatures of the GC injector or oven. Consequently, a two-step sequence of methoximation to derivatize ketones, followed by silylation to derivatize alcohols and carboxylic acids was performed. Thus, 2 mL of the total foot odor extract and active fractions 66–67, and 72–80 were transferred to 2 mL vials, concentrated just to dryness with a gentle flow of N_2_, and treated with 10 µL of a 20 mg/mL solution of methoxyamine HCl in dry pyridine. The resulting solutions were sonicated for 30 min at 23 °C, and transferred to an orbital shaker set to 100 rpm and 40 °C, for another 30 min. The resulting solutions were then treated with 50 µL of N,O-bis(trimethylsilyl)trifluoroacetamide (BSTFA, Sigma-Aldrich, Burlington MA, USA) and again placed in the shaker at 100 rpm and 40 °C for 1 h. The solutions were then diluted with 200 µL of dichloromethane and injected directly into the GC–MS.

Analyses of the derivatized extracts showed several peaks which were tentatively identified by interpretation of MS fragmentation patterns, matches with database spectra (W8N05ST; Wiley version 8.0 and NIST, version 5.0), and matches with published retention indices. Identifications were confirmed by matching retention times and mass spectra with those of authentic standards derivatized by the same procedure.

### Odor treatment reconstructed with authentic compounds

Synthetic compounds were initially tested as a blend of 2-ketoglutaric acid (2-KGA, 5 µg, 34 nmol) + lactic acid (5 µg, 55 nmol) or pyruvic acid (PA, 5 µg, 56 nmol) + lactic acid (5 µg, 55 nmol). The ratio of compounds used in the initial dose was based on the ratios found in the derivatized active fractions where each component was seen at an almost 1:1 ratio (Fig. 2 and S2). Solutions of various concentrations of each compound were made by serial dilution of a 1 mg/mL solution of each individual component of the mixture.10 µg/mL solutions of 2-KGA, LA, and PA were prepared for this experiment. 500 µL of each sample was evenly deposited dropwise on the surface of black glass beads in a 7-cm Petri dish as described earlier, while 1.0 mL of acetone control was deposited on an analogous Petri dish as a control. Both dishes were transferred to the hood and allowed to evaporate for 20 min, to ensure all solvent had evaporated. The Petri dishes were then transferred to the bioassay cage as previously described, with 9 replicates of each test. Each individual compound, as well as the 2-component (2-KGA + LA, and PA + LA) and 3-component (2-KGA + PA + LA) blends, was tested at the same doses the same protocol.

To determine the ratio-response of mosquitoes to the synthetic compounds, 2-KGA was tested at 5 µg (1:1), 500 ng (1:10), 50 ng (1:100), and 5 ng (1:1000), and 50 pg (1:100,000) combined with a constant amount of LA at 5 µg. PA was tested at 5 µg (1:1), 500 ng (1:10), 50 ng (1:100), 500 pg (1:10,000), 50 pg (1:100,000), and 500 fg (1:10,000,000) with a constant amount of LA at 5 µg. The dosage of lactic acid was kept constant because lactic acid is often found in large quantities in human sweat, and the ratio of lactic acid to pyruvic acid in sweat varies among individuals^25^.

To determine the importance of all three components as an orientation cue we tested a blend of 2-KGA, LA, and PA at a ratio of 1:0.01:1 (5 µg: 50 ng: 5 µg). This ratio was chosen due to the results of the ratio-response assays and literature reports of the natural ratio of LA to PA in human sweat^25^. Solutions of each sample were prepared as described above by serial dilution. We also tested a 2-component blend of PA + 2KGA to determine the importance of lactic acid in the mixture using the same ratio of PA:2-KGA found in the 3-component blend tested previously (0.01:1).

## Supplementary Information

Supplementary Information 1.

Supplementary Video 1.

Supplementary Video 2.

Supplementary Video 3.

Supplementary Video 4.

Supplementary Video 5.

Supplementary Video 6.

Supplementary Video 7.

Supplementary Video 8.

Supplementary Video 9.

Supplementary Video 10.

Supplementary Video 11.

Supplementary Video 12.

Supplementary Video 13.

## Data Availability

Raw bioassay results will be placed in https://github.com/bendemasisumner/compounds-from-human-odor-aedes-bello-carde.
