# Supplementary material for: Compounds from human odor induce attraction and landing in female yellow fever mosquitoes (Aedes aegypti)
Source: Sci Rep. 2022 Sep 21;12:15638. doi: 10.1038/s41598-022-19254-w (PMC9492692; doi:10.1038/s41598-022-19254-w)
Supplement: Supplementary file 1 — Supplementary Information 1. [file 41598_2022_19254_MOESM1_ESM.docx]

Supplemental Figures:


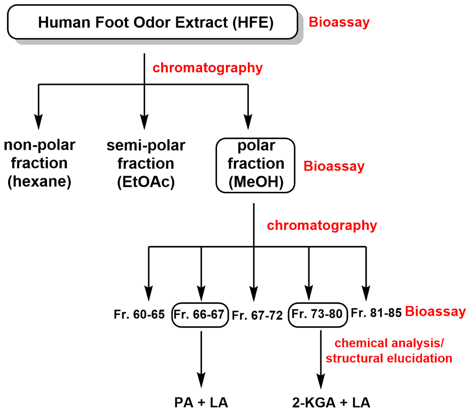


Figure S1. Stepwise fractionation and identification of active landing cue components using bioassay-guided fractionation. Circled fractions were the active fractions that fully or partially replicated the landing activity of the total human foot odor extract (HFE).


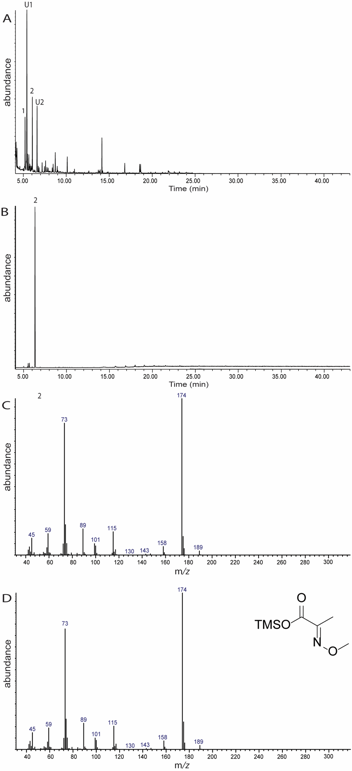


Figure S2. (A) GC chromatogram of derivatized fractions 66-67. (B) GC chromatogram of derivatized pyruvic acid synthetic standard. (C) Mass spectrum of compound 2. (D) Mass spectrum of derivatized pyruvic acid synthetic standard.

Figure S3. Landing response of host seeking *Ae. aegypti* to various odor treatments (n=9 per treatment). (A) Mean % landing responses of female *Ae. aegypti* to fractions 66-67 compared to solvent control. Total landing responses to fractions 66-67 were significant compared to landing responses on solvent control alone (p< 0.01). Here and in subsequent panels, asterisks indicate significance in landing choice differences between two treatments (*** = p <0.001, ** = p <0.01, * = p <0.05), error bars signify 95 % CI. (B) Mean % landing response of female *Ae. aegypti* to fractions 73-80 compared to solvent control. Total landing responses to fractions 73-80 were significant compared to landing responses on solvent control alone (p< 0.0001). (C) Mean % landing response of female *Ae. aegypti* to lactic acid (LA, 5 µg) alone compared to solvent control. Total landing response to lactic acid (5 µg) were significant compared to solvent alone but were comparatively less than the landing rates seen with the three component and two component blends (p < 0.01). (D) Mean % landing response of female *Ae. aegypti* to 2-ketoglutaric acid (2-KGA, 5 µg) alone compared to solvent control. Total landing responses to 2-KGA (5 µg) were significant compared to solvent alone but were comparatively much lower than the landing rates seen in the 3-component and 2-component blends (p < 0.01). (E) Mean % landing of female *Ae. aegypti* to pyruvic acid (PA, 50 ng) alone compared to solvent control. Total landing responses were not significant compared to control, indicating that pyruvic acid was not attractive alone. (F) Comparison of landing responses of three component 2-KGA + LA + PA blend to two component 2-KGA + LA and PA +LA treatments at optimal ratio doses using mixed model ANOVA with Tukey’s multiple comparison test. There was no significant difference between the 3-component blend and 2-component 2-KGA + LA blend, indicating that all three components were not necessary for the increased landing attraction.

Videos:

Video S1. Video recording of mosquito cage landing assays. Treatments: human foot odor (left) vs. clean control beads (right). Twenty-seven host-seeking female *Ae. aegypti* were released into the cage and their landings were scored every 30 seconds for a 6-minute interval. This recording shows the 30 sec-1 min 27sec interval of one replicate.

Video S2. Video recording of mosquito cage landing assays. Treatments: acetone extract of human foot odor (HFE, right) vs. acetone treated control beads (left). Twenty-eight host-seeking female *Ae. aegypti* were released into the cage and their landings were scored once every 30 seconds for a 6-minute interval. This recording shows the 30 sec-1 min 44 sec interval of one replicate.

Video S3. Video recording of mosquito cage landing assay. Treatments: the non-polar (pentanes) fraction (left) vs. pentanes treated control beads (right). Twenty-eight host-seeking female *Ae. aegypti* were released into the cage and their landings were scored over a 6-minute interval. This recording shows the 30 sec-1 min 14 sec interval of one replicate.

Video S4. Video recording of mosquito cage landing assays, Treatments: the semi-polar (ethyl acetate) fraction (right) vs. EtOAc treated control beads (left). Twenty-eight host-seeking female *Ae. aegypti* were released into the cage and their landings were scored over a 6-minute interval. This recording shows the 1 min -1 min 37 sec interval of one replicate.

Video S5. Video recording of mosquito cage landing assays. Treatments: the polar (MeOH) fraction (right) vs. MeOH treated control beads (left). Twenty-nine host-seeking female *Ae. aegypti* were released into the cage and their landing behaviors were recorded over a 6-minute interval. This recording shows the 00:30 – 01:23 interval of one replicate.

Video S6. Video recording of mosquito cage landing assays. Treatments: fraction 66-67 (right) vs. MeOH treated control beads (left). Twenty-nine host-seeking female *Ae. aegypti* were released into the cage and their landing behaviors were recorded over a 6-minute interval. This recording shows the 01:00 – 02:08 min interval of one replicate.

Video S7. Video recording of mosquito cage landing assays. Treatments: Fraction 73-80 (right) vs. MeOH treated control beads (left). Twenty-eight host-seeking female *Ae. aegypti* were released into the cage and their landing behaviors were recorded over a 6-minute interval. This recording shows the 00:30 – 02:17 min interval of one replicate.

Video S8. Video recording of mosquito cage landing assays. Treatments: pyruvic acid (PA, 5 µg, 56 nmol) + lactic acid (LA, 5 µg, 54 nmol) (left Petri dish) vs. acetone treated control beads (right). Thirty-two host-seeking female *Ae. aegypti* were released into the cage and their landing behaviors were recorded over a 6-minute interval. This recording shows the 01:00 – 02:01 min interval of one replicate.

Video S9. Video recording of mosquito cage landing assays. Treatments: 2-ketoglutaric acid (2-KGA, 5 µg, 34 nmol) + lactic acid (LA, 5 µg, 54 nmol) (left Petri dish) vs. acetone treated control beads (right). Twenty-seven host-seeking female *Ae. aegypti* were released into the cage and their landing behaviors were recorded over a 6-minute interval. This recording shows the 01:00 – 02:34 min interval of one replicate.

Video S10. Video recording of mosquito cage landing assays. Treatments: lactic acid (LA, 5 µg, 54 nmol) (right Petri dish) vs. acetone treated control beads (left). Thirty-two host-seeking female *Ae. aegypti* were released into the cage and their landing behaviors were recorded over a 6-minute interval. This recording shows the 00:30- 01:34 min interval of one replicate.

Video S11: Video recording of mosquito cage landing assays. Treatments: PA+LA+2KA (5µg: 50 ng: 5µg) (left Petri dish) vs. acetone treated control beads (right). Twenty-eight host-seeking female *Ae. aegypti* were released into the cage and their landing behaviors were recorded over a 6-minute interval. This recording shows the 00:30- 01:37 min interval of one replicate.

Video S12: Video recording of mosquito probing 2-KGA+LA treatments.
